# Influence of the Workplace on Influenza and COVID-19 Vaccination Acceptance Among Swiss Healthcare Workers During Season 2021/22

**DOI:** 10.3389/ijph.2026.1608922

**Published:** 2026-05-08

**Authors:** Olga Morgel, Astrid Czock, Jan Fehr, Phung Lang

**Affiliations:** 1 LongevityDoc GmbH, Weingartenstrasse, Rüschlikon, Switzerland; 2 Czock Healthcare Consulting, Talstrasse, Davos, Switzerland; 3 University of Zurich, Epidemiology, Biostatistics and Prevention Institute, Zurich, Switzerland

**Keywords:** COVID-19, HCWs, influenza, vaccination acceptance, vaccination rate

## Abstract

**Objectives:**

Healthcare workers (HCWs) are essential in preventing and controlling infectious diseases and can influence public trust in vaccines. This study compares vaccination behaviors among HCWs in a Swiss hospital setting with those in primary care and identifies key factors influencing vaccine acceptance.

**Methods:**

An online questionnaire was distributed to HCWs at a cantonal hospital in Central Switzerland. The results were compared with a previous nationwide survey of German-speaking HCWs in primary care using descriptive statistics, chi-square tests, and multivariable logistic regression analyses.

**Results:**

Influenza vaccination coverage was 37.2% in hospitals and 59.8% in primary care. COVID-19 vaccination rates were high in both settings (89.1% and 92.7%, respectively). Physicians and older HCWs were significantly more likely to be vaccinated than nurses and younger staff. Vaccination training and prior vaccination history were associated with higher vaccination rates and stronger recommendation behavior. The most common reasons supporting vaccination were self-protection and patient protection.

**Conclusion:**

Vaccination behavior among HCWs differs considerably by healthcare setting and profession. Targeted vaccination training, particularly for younger HCWs and nurses in hospital settings, may help increase vaccine acceptance.

## Introduction

Vaccination is a vital public health intervention, especially for combating infectious diseases such as seasonal influenza and COVID-19. Healthcare workers (HCWs) play a pivotal role in mitigating the impact of these infections through vaccination. Being at the forefront of patient care, they are exposed to or affected by infections. Given the significant implications for healthcare systems and public health, vaccination acceptance rates in HCWs and factors influencing these rates are of paramount importance [1–3].

Seasonal influenza is one of the most common causes of acute respiratory infections worldwide. According to the World Health Organization (WHO), influenza causes an estimated 1 billion infections annually worldwide, of which about 3–5 million are severe cases resulting in up to 650,000 deaths [4]. In Switzerland, the annual incidence of influenza cases varies, but it is estimated that the disease leads to thousands of hospitalizations and several hundred deaths each year, particularly among vulnerable populations such as the elderly and individuals with pre-existing conditions [5].

Since the start of the COVID-19 pandemic in 2019, over 600 million people have been infected and more than 6 million have died from the virus worldwide. In Switzerland, as of July 2024, over 4 million COVID-19 cases and more than 12,000 associated deaths have been reported [6]. Even after the introduction and widespread availability of COVID-19 vaccines, the disease remains a significant health risk, particularly for vulnerable populations and in the context of ongoing community transmission [7].

While hospital and primary care (PC) settings differ in dynamics and patient demographics, HCWs in both settings face a similarly increased risk of exposure to influenza [8, 9] and COVID-19 [10]. Knowledge, awareness, access to information, and peer influence are important potential influencing factors [11–15]. As HCWs influence public trust and vaccine acceptance rates [16, 17] it is essential to understand their vaccination behaviors in order to address setting-specific barriers. The study aimed to identify factors associated with vaccination uptake and to compare vaccination behaviors among Swiss HCWs in PC with those in the hospital settings, with the goal of informing targeted public health strategies to strengthen healthcare system resilience against current and future infectious disease threats.

## Methods

### Data Collection

This observational, cross-sectional study was conducted in the winter of 2021/22. For comparability, data collection method, questionnaire and analysis were analogous to the nationwide study conducted during the same season by Morgel et al, which evaluated factors associated with influenza and COVID-19 vaccination uptake among HCW working in PC settings [18].

Briefly, the national study by Morgel et al. [18] was conducted in French, Italian and German to cover all regions of Switzerland (to avoid confusion, this study will be referred as PC study). Invitations to participate were distributed via newsletters from various healthcare stakeholders with a focus on primary care settings, including national professional societies, healthcare leagues and medical networks to their members. The targeted population comprised HCWs from multiple profession, including physicians, nurses, pharmacists, medical practice assistants, pharmacy technicians and public health officers.

This present survey was conducted exclusively in German, but targeted the same groups of HCWs as listed above. Participants were recruited from one hospital in Central Switzerland and were invited per email to complete a questionnaire. The survey included HCWs from the following departments and divisions: surgery; internal medicine; clinical cross-sectional medicine (anesthesia, emergency medicine, operating room management, intensive care medicine); specialized clinics and institutes (laboratory medicine, pathology, pharmacy, radio-oncology, radiology, and nuclear medicine); as well as nursing and social care services. In total, 4,727 HCWs received an electronic invitation to participate in the study with a link to an online questionnaire, which they could answer during 2 months.

Based on an assumption of 30% influenza vaccination uptake, 5% precision, a population size of 4727, a sample size of 302 participants was needed for the study.

No ethical approval was required (Basec No. 2021-01316). Participation was voluntary and collected data were fully anonymized, with a positive response implying consent for an anonymized analysis.

### Questionnaire

To ensure a direct comparison of the collected data, identical questions were asked to HCWs in both PC and hospital settings. The questionnaire was created in German by the QualiCCare working group for the study published in 2024 [18].

The questionnaire was structured into three sections, beginning with the demographics (age, sex, and profession), followed by questions concerning influenza vaccination, including vaccination status and history, reasons for either receiving or foregoing vaccination, and vaccination recommendation behavior. Finally similar questions were asked regarding COVID-19, also inquiring on timing and number of COVID-19 vaccine doses, vaccine type (mRNA-based or otherwise), and possible COVID-19 infections. Participants were also asked about their vaccination training status, including whether they were authorized to administer vaccines, were currently in training, or plan to pursue such training in the future. The questions aimed to capture the practical aspect of vaccination administration, including qualification and professional role in the vaccination process.

Participants were given a link to the online platform, SurveyMonkey to participate in the study. In line with data protection protocol, IP-tracking was disabled, rendering submitted questionnaires irrevocable.

The online questionnaire was accessible to HCWs in the PC from November 2021 to February 2022, and to hospital staff in January/February 2022. Two reminders were sent to encourage participation in the PC survey and one in the hospital survey.

### Analysis

For comparative purposes, the German-language subset of the data from the PC study [18] was extracted and re-analyzed. Data from the PC study and this study (hospital data) were categorized by occupation, sex, age, vaccination training status, vaccination status and history, motivations for and against vaccination, frequency of recommending vaccinations, and reasons for getting vaccinated against influenza and COVID-19. Only responses with complete datasets were included in the analysis. Pearson chi-square tests of independence were performed to explore the relationship between influenza vaccination status and various factors such as HCW occupation, age, and their likelihood to recommend the influenza vaccine (and similarly for COVID-19). Additionally, 2 × 2 contingency tables were used to compare the PC and hospital survey data. Crude and adjusted odds ratios (ORs and AORs), with 95% confidence intervals (CIs), were estimated using logistic regression. Multivariable models were adjusted for sex, age group, profession, and vaccination training history to evaluate factors associated with the likelihood of receiving an influenza or COVID-19 vaccine. p-value <0.05 was considered significant. All data analysis was performed using Stata software (version 17.0).

## Results

### Participant Characteristics

In the hospital setting, the survey was distributed via email to 4,727 recipients. A total of 1,215 responses were received, corresponding to a response rate of 25.7%. After data cleaning, 1,140 questionnaires were retained for analysis, yielding a completion rate of 93.9%. 75 responses were excluded due to incomplete answers.

From the PC study [18] 834 German questionnaires were re-analyzed. Because the survey link was disseminated through channels for which the total number of recipients could not be determined, the response rate for the PC setting could not be calculated.

Participation results can be found in Tables 1–3, with Table 1 showing participation by profession, Table 2 by sex distribution and Table 3 by age groups. Participation in the hospital setting was highest among nurses (52.7%) and lowest among pharmacists (1.1%) and pharmacy technicians (0.4%); in PC, it was lowest among nurses (5.8%) and highest among pharmacists (33.9%) and medical practice assistants (MPAs, 21.3%) (Table 1). Physicians’ participation was similar in both settings: Hospital (19.1%) and PC (18.2%).

**TABLE 1:** Vaccination rates and recommendation for influenza and COVID-19 vaccinations among healthcare workers in primary care and hospital, detailed by profession, vaccination training and influenza season (Switzerland, 2021/22).

Categories	Profession, n (%)							
Settings	Physician	Pharmacist	Nurse	MPA	Pharmacy Technician	Others^a^ ^,^ ^b^	Total, n	p-value
**Demographic distribution**								
Primary care_de	152 (18.2)	283 (33.9)	48 (5.8)	178 (21.3)	50 (6.0)	123 (14.8)	834	
Hospital	218 (19.1)	12 (1.1)	601 (52.7)	44 (3.9)	4 (0.4)	261 (22.9)	1,140	-
*Vaccination training* ^c^								
Primary care_de	136 (89.5)	202 (71.4)	22 (45.8)	159 (89.3)	14 (28.0)	13 (10.6)	546 (65.5)	<0.001
Hospital	101 (46.3)	3 (25.0)	381 (63.4)	28 (63.6)	1 (25.0)	45 (17.2)	559 (49.0)	<0.001
**Vaccination uptake**								
*Influenza vaccinated*								
Primary care_de	131 (86.2)	211 (74.5)	22 (45.8)	91 (51.1)	21 (42.0)	23 (18.7)	499 (59.8)	<0.001
Hospital	162 (74.3)	12 (100)	161 (26.8)	8 (18.2)	0	81 (31.0)	424 (37.2)	<0.001
*Recommend influenza vaccination* ^d^								
Primary care_de	102 (67.1)	226 (92.6)	27 (56.3)	129 (72.5)	24 (48.0)	28 (22.8)	536 (64.3)	<0.001
Hospital	146 (67.0)	5 (41.7)	185 (30.8)	11 (25.0)	0	53 (20.3)	400 (35.1)	<0.001
*Influenza 2021/22* ^e^ *I plan to get vaccinated*								
Primary care_de	131 (86.2)	210 (74.2)	21 (43.8)	76 (42.7)	20 (40.0)	27 (22.0)	485 (58.2)	<0.001
Hospital	163 (74.8)	11 (91.7)	133 (22.1)	7 (15.9)	0	69 (26.4)	383 (33.6)	<0.001
*COVID-19 2020/21 * *vaccinated*								
Primary care_de	145 (95.4)	271 (95.8)	42 (87.5)	160 (89.9)	44 (88.0)	111 (90.3)	773 (92.7)	0.032
Hospital	210 (96.3)	12 (100)	516 (85.9)	37 (84.1)	4 (100)	237 (90.8)	1,016 (89.1)	<0.001
*Recommend COVID-19 2020/21 vaccination* ^d^								
Primary care_de	119 (78.3)	256 (90.5)	33 (68.8)	147 (82.6)	34 (68.0)	48 (39.0)	637 (76.4)	<0.001
Hospital	193 (88.5)	6 (50.0)	360 (59.9)	24 (54.5)	0	100 (38.3)	683 (59.9)	<0.001

n = number of participants. Total primary care_all n = 1,237. Total primary care_de n = 834. Total in hospital setting n = 1,141. Primary care (PC) data [18]. Hospital = H. Primary care_de–German-speaking participants of primary care setting.

^a^ Others Profession in PC: psychosocial therapist, administration, public health specialist, social worker, other healthcare profession.

^b^ Others Profession in H: Bioanalytic, emergency service, psychosocial therapist, administration, social worker, other healthcare profession.

^c^ For the variable vaccination training, there were 4 possible answers (yes, no, I am in training now, I am thinking about it), but only the responses for “yes” are displayed.

^d^ For the variable recommend, there were 4 possible answers (yes, no, no patients, only in certain cases), but only the responses for “yes” are displayed. p-values are calculated for all 4 possible answers.

^e^ For the variable Influenza 2021/22, there were 3 possible answers (yes, no, do not know), but only the responses for “yes” are displayed. p-values are calculated for all 3 possible answers.

**TABLE 2:** Vaccination rates and recommendation for influenza and COVID-19 vaccinations among healthcare workers in primary care and hospital detailed by sex and vaccination training (Switzerland, 2021/22).

Categories	Sex			p-value	Total, n
Settings	f	m	d		
**Demographic distribution**					
Primary care_de	698 (83.7)	132 (15.8)	4(0.48)		834
Hospital, n (%)	909 (79.7)	229 (20.1)	2 (0.2)	-	1,140
Swiss population 2021, % [**]	50.3	49.7	-		8,738,791
*Vaccination training* ^a^					
Primary care_de	462 (66.2)	84 (63.6)	-	0.025	546 (65.5)
Hospital, n (%)	448 (49.2)	110 (48.0)	1 (50.0)	0.749	559 (49.0)
	Pearson chi2(2) = 5.7124 Pr = 0.057				
**Vaccination uptake**					
*Influenza vaccinated*					
Primary care_de	401 (57.5)	96 (72.7)	2 (50.0)	0.004	499 (59.8)
Hospital, n (%)	278 (30.5)	145 (63.3)	1 (50.0)	<0.001	424 (37.2)
	Pearson chi2(2) = 42.9741 Pr < 0.001				
*Recommend influenza vaccination* ^b^					
Primary care_de	450 (64.5)	85 (64.4)	1 (25.0)	0.216	536 (64.3)
Hospital, n (%)	283 (31.1)	117 (51.1)	0	<0.001	400(35.1)
	Pearson chi2(2) = 35.7168 Pr < 0.001				
*Influenza 2021/22* ^c^ *I plan to get vaccinated*					
Primary care_de	385 (55.2)	98 (74.2)	2 (50.0)	0.001	485 (58.2)
Hospital, n (%)	247 (27.2)	136 (59.4)	0	<0.001	383 (33.7)
	Pearson chi2(2) = 37.9406 Pr < 0.001				
*COVID-19 2020/21 vaccinated*					
Primary care_de	646 (92.6)	123 (93.2)	4 (100)	0.826	773 (92.7)
Hospital, n (%)	795 (87.5)	219 (95.6)	2 (100.0)	<0.001	1,016 (89.1)
	Pearson chi2(2) = 15.3273 Pr < 0.001				
*Recommend COVID-19 2020/21 vaccination* ^b^					
Primary care_de	537 (76.9)	98 (74.2)	2 (50.0)	0.155	637 (76.4)
Hospital, n (%)	509 (55.9)	173 (75.5)	1 (50.0)	<0.001	683 (59.9)
	Pearson chi2(2) = 31.2518 Pr < 0.001				

n = number of participants. Primary care_de – German speaking participants of primary care setting. Primary care data [18]. [**]Swiss Federal Statistical Office. Swiss Population by Language 2020 [Internet]. Bern; 2020 [cited 2025 Sep 17]. Available from: https://www.bfs.admin.ch/bfs/de/home/ statistiken/ bevoelkerung/sprachen-religionen.assetdetail.21344054.html [**] Swiss Federal Statistical Office. Swiss Population 2021 [Internet]. Bern; 2021 [cited 2025 Sep 17]. Available from: https://www.bfs.admin.ch/bfs/de/home/statistiken/bevoelkerung/erhebungen/bevnat.html.

&p-value for sex only compares values between males (m) and females (f) as the n for undefined gender (d) is too small.

^a^ For the variable vaccination training, there were 4 possible answers (yes, no, I am in training now, I am thinking about it), but only the responses for “yes” are displayed.

^b^ For the variable recommend, there were 4 possible answers (yes, no, no patients, only in certain cases), but only the responses for “yes” are displayed. p-values are calculated for all 4 possible answers, using Fischer exact test.

^c^ For the variable Influenza 2021/22, there were 3 possible answers (yes, no, do not know), but only the responses for “yes” are displayed. p-values are calculated for all 3 possible answers.

**TABLE 3:** Vaccination rates and recommendation for influenza and COVID-19 vaccinations among healthcare workers in primary care and hospital, detailed by age group and influenza season (Switzerland, 2021/22).

Categories	Age of the participants						Total	p-value
Settings	16–20	21–30	31–40	41–50	51–60	61+	n	
**Demographic distribution**								
Primary care_de	19 (2.3)	86 (10.3)	159 (19.1)	229 (27.5)	261 (31.3)	80 (9.6)	834	
Hospital, n (%)	85 (7.5)	295 (25.8)	301 (26.4)	218 (19.1)	197 (17.3)	44 (3.9)	1,140	-
Swiss Population 2021[**],%	4.8	12.1	14.3	13.7	14.7	9.6	8,738,791	
*Vaccination Training* ^a^								
Primary care_de	11 (57.9)	60 (69.8)	114 (71.7)	138 (60.3)	172 (65.9)	51 (63.8)	546 (65.5)	0.063
Hospital, n (%)	49 (57.6)	178 (60.3)	146 (48.5)	91 (41.7)	77 (39.1)	18 (41.0)	559 (49.0)	<0.001
**Vaccination uptake**								
*Influenza vaccinated*								
Primary care_de	4 (21.1)	39 (45.4)	90 (56.6)	136 (59.4)	177 (67.8)	53 (66.3)	499 (59.8)	<0.001
Hospital, n (%)	15 (17.7)	92 (31.2)	123 (40.9)	89 (40.8)	82 (41.6)	23 (52.3)	424 (37.2)	<0.001
Swiss Population [**], %	-	-	-	-	-	38	-	
Swiss HCW [@@], %	-	-	-	-	-	-	26	
*Recommend influenza vaccination* ^b^								
Primary care_de	9 (47.4)	63 (73.3)	94 (59.1)	136 (59.4)	179 (68.6)	55 (68.8)	536 (64.3)	0.017
Hospital, n (%)	16 (18.8)	106 (35.9)	107 (35.5)	72 (33.0)	79 (40.1)	20 (45.5)	400 (35.1)	<0.001
*Influenza 2021/22* ^c^ *I plan to get vaccinated*								
Primary care_de	3 (15.8)	32 (37.2)	84 (52.8)	132 (57.6)	181 (69.4)	53 (66.3)	485 (58.2)	<0.001
Hospital, n (%)	7 (8.2)	85 (28.8)	113 (37.5)	83 (38.1)	70 (35.5)	25 (56.8)	383 (33.6)	<0.001
*COVID-19 2020/21 vaccinated*								
Primary care_de	16 (84.2)	74 (86.1)	147 (92.5)	214 (93.5)	248 (95.0)	74 (92.5)	773 (92.7)	0.078
Hospital, n (%)	68 (80.2)	260 (88.1)	263 (87.4)	198 (90.8)	184 (93.4)	43 (97.7)	1,016 (89.1)	0.006
Swiss Population [@], %	49.2	71.5	73.2	77.3	80.5	86.1	70	
Swiss HCW [@@], %							85.0/+5.0	
*Recommend COVID-19 2020/21 vaccination* ^b^								
Primary care_de	10 (52.6)	67 (77.9)	117 (73.6)	166 (72.5)	213 (81.6)	64 (80.0)	637 (76.4)	0.088
Hospital, n (%)	41 (48.2)	171 (58.0)	187 (62.1)	127 (58.3)	126 (64.0)	31 (70.5)	683 (59.9)	<0.001

n = number of participants. Primary care_de–German speaking participants of primary care setting. Primary care_all–all participants of primary care setting. [@] Vaccination Data from Swiss Federal Health Office (BAG) Bericht zur Grippesaison 2020/21 BAG-Bulletin, Page 7. https://www.bag.admin.ch/dam/bag/de/dokumente/mt/infektionskrankheiten/grippe/saisonbericht-grippe-2020-21.pdf.download.pdf/saisonbericht-grippe-2020-21.pdf. [Accessed January 22, 2026]. Primary care data [18]. [**] Population data from Swiss Federal Statistical Office 2015/16 et 2020/21 https://www.bfs.admin.ch/bfs/en/home/statistics/population.html [Accessed January 22, 2026 [@] Vaccination Data from Swiss Federal Health Office (BAG) COVID-⁠19 Switzerland | Coronavirus | Dashboard (admin.ch) [Accessed January 22, 2026]. [@@] Vaccination Data from FMH Study November 2021. https://www.fmh.ch/files/pdf26/hohe-impfbereitschaft-bei-aerztinnen-und-aerzten.pdf [Accessed January 22, 2026].

^a^ For the variable vaccination training, there were 4 possible answers (yes, no, I am in training now, I am thinking about it), but only the responses for “yes” are displayed.

^b^ For the variable recommend, there were 4 possible answers (yes, no, no patients, only in certain cases), but only the responses for “yes” are displayed. p-values are calculated for all 4 possible answers, using Chi2 test.

^c^ For the variable Influenza 2021/22, there were 3 possible answers (yes, no, do not know), but only the responses for “yes” are displayed. p-values are calculated for all 3 possible answers.

The sex distribution was somewhat similar in both settings, with more females (80%–84%) than males (16%–20%) participating (Table 2). Distribution by age groups showed higher participation of young HCWs 21–40 years in the hospital (Table 3).

### Vaccination Status by Profession, Age and Sex

Results of vaccination uptake for both influenza and COVID-19 can be found in Tables 1–3, with Table 1 showing vaccination uptake by profession, Table 2 by sex distribution and Table 3 by age groups. Multivariable logistic regression models used for exploratory analyses can be found in Supplementary Table 1a for influenza and Supplementary Table 1b for COVID-19.

#### Influenza Vaccination Status

Influenza vaccination rates among HCWs varied between settings in 2021/22 with 59.8% in PC and 37.2% in hospitals (Table 1). Vaccination acceptance was higher among physicians in both PC (86.2%) and hospitals (74.3%), compared to nurses (45.8% in PC, 26.8% in hospitals). After controlling for sex, age group, profession and vaccination training history, adjusted odds ratios (AOR) indicated physicians and pharmacists in PC were significantly more likely to be vaccinated than nurses (AOR: 5.5 and 3.4, respectively, p < 0.001) (Supplementary Table 1b). In hospitals, physicians and other professions had higher vaccination odds compared to nurses (AOR: 6.5, p < 0.001 and AOR: 1.5, p = 0.026, respectively).

Future vaccination intentions also differed, with 58.2% HCWs in PC and 33.6% in hospitals planning to vaccinate against influenza (Table 1). Physicians showed the highest intent (PC - 86.2%, Hospital - 74.8%), while nurses and MPAs in hospitals were least likely (22.1% and 15.9%, respectively) compared to colleagues in PC (43.8% and 42.7%, respectively).

Sex significantly influenced vaccination rates, with males showing higher rates than females in both settings (p-value <0.001 in PC, 0.004 in Hospital, Table 2). Female HCWs in PC had higher vaccination rates (57.5%) compared to their hospital counterparts (30.5%). Multivariable logistic regression showed female HCWs in hospitals have significantly lower odds of being vaccinated than their male counterpart (AOR: 0.5, p < 0.001, Supplementary Table 1a), while there was no difference in PC (AOR: 1.4, p = 0.409).

Age trends showed older HCWs had higher influenza vaccination rates, with significant differences across age groups (p < 0.001, Table 3). The likelihood of vaccination increased with age, particularly in PC where older age groups had significantly higher odds (Table 3; Supplementary Table 1a AOR 4.5, p =0.014) compared to the 16–20 age group. In the hospital setting, rates increased from 17.7% among the youngest to 52.3% in the oldest group (Table 3; Supplementary Table 1a, AOR 2.6, p = 0.035). The intention to get vaccinated followed the same pattern (Table 3).

#### COVID-19 Vaccination Status

In both settings, HCWs reported high COVID-19 vaccination rates, with 92.7% (range 88%–95%) in PC and 89.1% (range 84%–100%) in the hospital (Table 1). Compared to nurses, only physicians were significantly more likely to be vaccinated in the hospital setting (AOR: 3.1, p = 0.005, Supplementary Table 1b) while it was pharmacists in the PC setting (AOR: 3.4, p = 0.026, Supplementary Table 1b).

The disparity between the sexes was evident only in the hospital, where female HCWs were significantly less likely to be vaccinated than males (Table 2; Supplementary Table 1b, AOR: 0.5, p =0.045).

Age analysis revealed a consistent trend across both settings, with older HCWs displaying higher vaccination rates (Table 3, PC p =0.078; hospital p =0.006). Specifically, those aged 51–60 in hospitals were significantly more likely to be vaccinated (Supplementary Table 1b, AOR: 2.9, p =0.009).

### The Impact of Vaccination Status and History on the Likelihood of Recommending Vaccination and the Intention to Get Vaccinated

Overall, 64.3% of HCWs in PC and 35.1% in the hospital settings recommended influenza vaccination while it was 76.4% and 59.9%, respectively, for COVID-19 vaccination (Tables 1–3). More specifically, HCWs who had been vaccinated were more likely to recommend the influenza or COVID-19 vaccine (Table 4). Unvaccinated HCWs showed a higher tendency to recommend the vaccine only in certain cases or not at all (Table 4). Among 499 (59.8%) influenza vaccinated PC HCWs, only 0.6% never recommended the influenza vaccine, while 79.2% recommended it. A similar trend was observed in hospitals: among 424 (37.2%) vaccinated HCWs, 4.5% did not recommend the vaccine while 59.4% recommended it, with 86 (20.3%) advising it in certain scenarios.

**TABLE 4:** Recommendations of influenza and COVID-19 vaccinations by healthcare workers in the primary care and hospital settings, detailed by vaccination status and level of vaccination training (Switzerland, 2021/22).

Vaccination and training status			Vaccination recommendation status							
	Total, n		No		Yes		No patients		In certain cases	
	PC	H	PC	H	PC	H	PC	H	PC	H
Influenza vaccinated 2020/21			Recommend the influenza vaccine, n (%)							
No	335	716	22 (6.6)	126 (17.6)	141 (42.1)	148 (20.7)	77 (23.0)	174 (24.3)	95 (28.4)	268 (37.4)
Yes	499	424	3 (0.6)	19 (4.5)	395 (79.2)	252 (59.4)	23 (4.6)	67 (15.8)	78 (15.6)	86 (20.3)
Total, n	834	1,140	25 (3.0)	145 (12.7)	536 (64.3)	400 (35.1)	100 (12.0)	241 (21.1)	173 (20.7)	354 (31.1)
COVID-19 vaccinated 2020/21			Recommend the COVID-19 vaccine, n (%)							
No	61	124	12 (19.7)	55 (44.4)	11 (18.0)	9 (7.3)	13 (21.3)	27 (21.8)	25 (41.0)	33 (26.6)
Yes	773	1,016	12 (1.6)	55 (5.0)	626 (81.0)	674 (66.3)	81 (10.5)	182 (17.9)	54 (7.0)	109 (10.7)
Total, n	834	1,140	24 (2.9)	106 (9.3)	637 (76.4)	683 (59.9)	94 (11.3)	209 (18.3)	79 (9.5)	142 (12.5)
Vaccination training^a^			Recommend the influenza vaccine, n (%)							
No	281	581	15 (5.3)	71 (12.2)	117 (41.6)	173 (29.8)	80 (28.5)	181 (31.1)	69 (24.6)	156 (26.9)
Yes	553	559	10 (1.8)	74 (13.2)	419 (75.8)	227 (40.6)	20 (3.6)	60 (10.7)	104 (18.8)	198 (35.4)
Total, n	834	1,140	25 (3.0)	145 (12.7)	536 (64.3)	400 (35.1)	100 (12.0)	241 (21.1)	173 (20.7)	354 (31.1)
Vaccination training^a^			Recommend the COVID-19 vaccine, n (%)							
No	281	581	14 (5.0)	51 (8.8)	164 (58.4)	304 (52.3)	75 (26.7)	160 (27.5)	28 (10.0)	66 (11.4)
Yes	553	559	10 (1.8)	55 (9.8)	473 (85.5)	379 (67.8)	19 (3.4)	49 (8.8)	51 (9.2)	76 (13.6)
Total, n	834	1,140	24 (2.9)	106 (9.3)	637 (76.4)	683 (59.9)	94 (11.3)	209 (18.3)	79 (9.5)	142 (12.5)

Setting: PC, primary care, but only German speaking participants, H - hospital. n = number of participants. PC Total n = 834; H Total n = 1,140 (%) = Percent of participants; no patients = no contact with patients. Primary care data [18].

^a^ For the variable vaccination training, there were 4 possible answers (yes, no, I am in training now, I am thinking about it), but only the responses for “yes” and “no” are displayed. The group “I am in training now” was added to the “yes”. The group “I am thinking about it” to “no”.

COVID-19 vaccination trends were similar in both settings (Table 4). Among 773 (92.7%) COVID-19 vaccinated HCWs in PC, only 1.6% never recommend the COVID-19 vaccine, while 81.0% recommended it. Among 1,016 (89.1%) COVID-19 vaccinated HCWs in the hospital setting, 5.0% never recommend the vaccine, while 66.3% consistently recommend it.

Similarly, 90.6% of those who were vaccinated during 2020/21 against the influenza in the PC setting and 78.8% in the hospital setting expressed the intention to be vaccinated against influenza in 2021/22, compared with 9.9% of HCWs in the PC group and 7.0% in the H group who were unvaccinated (Table 5, PC: chi2(1) = 539.4445, Pr < 0.001): Hospital: (chi2(2) = 624.1118, Pr < 0.001).

**TABLE 5:** Vaccination status of healthcare workers related to level of vaccination training and vaccination history (Switzerland, 2021/22).

A Training status^a^	Influenza vaccination, n (%)					
	Yes		NO		Total	
	PC	H	PC	H	PC	H
Yes	388 (70.2)	223 (52.6)	165 (29.8)	336 (46.9)	553 (66.3)	559 (49.0)
No	111 (39.5)	201 (47.4)	170 (60.5)	380 (53.1)	281 (33.7)	581 (51.0)
Total	499 (59.8)	424 (37.2)	335 (40.2)	716 (62.8)	834 (100)	1,140 (100)
	PC: Pearson chi2(1) = 72.8826 Pr < 0.001 H: Pearson chi2(1) = 3.5036 Pr = 0.061					

B Training status^a^	COVID-19 vaccination, n (%)					
	Yes		No		Total	
	PC	H	PC	H	PC	H
Yes	520 (94.0)	490 (87.7)	33 (6.0)	69 (12.3)	553 (66.3)	559 (51.0)
No	253 (90.0)	526 (90.5)	28 (10.0)	55 (9.5)	281 (33.7)	581 (49.0)
Total	773 (92.7)	1,016 (89.1)	61 (7.3)	124	834 (100)	1,140 (100)
	PC: Pearson chi2(1) = 4.3908 Pr = 0.036 H: Pearson chi2(1) = 2.4641 Pr = 0.116					

C Influenza vaccinated 2020/21	Intention to get the influenza vaccination 2021/22, n (%)					
	Yes		No		Do not know	
	PC	H	PC	H	PC	H
Yes	452 (90.6)	334 (78.8)	34 (6.8)	56 (13.2)	13 (2.6)	34 (8.0)
No	33 (9.9)	50 (7.0)	259 (77.3)	549 (76.6)	43 (12.8)	118 (16.5)
Total	485 (58.2)	384 (33.7)	293 (35.1)	605 (53.0)	56 (6.7)	152 (13.3)
	PC: Pearson chi2(1) = 539.4445 Pr < 0.001 H: Pearson chi2(2) = 624.1118 Pr < 0.001					

D COVID-19 vaccinated 2020/21	Intention to get the influenza vaccination 2021/22, n (%)					
	Yes		No		Do not know	
	PC	H	PC	H	PC	H
Yes	479 (62.0)	383 (37.7)	241 (31.2)	487 (47.9)	53 (6.9)	147 (14.5)
No	6 (9.8)	1 (0.8)	52 (85.2)	118 (95.2)	3 (4.9)	5 (4.0)
Total	485 (58.2)	384 (33.7)	293 (35.1)	605 (53.0)	56 (6.7)	152 (13.3)
	PC: Pearson chi2(1) = 73.7844 Pr < 0.001 H: Pearson chi2(2) = 100.2009 Pr < 0.001					

E Influenza vaccinated 2020/21	COVID-19 vaccination, n (%)					
	Yes		No		Total	
	PC	H	PC	H	PC	H
Yes	493 (98.8)	422 (99.5)	6 (1.2)	2 (0.5)	499 (59.8)	424 (37.2)
No	280 (83.6)	594 (83.0)	55 (16.4)	122 (17.0)	335 (40.2)	716 (62.8)
Total	773 (92.7)	1,016 (89.1)	61 (7.3)	124 (10.9)	834 (100)	1,140 (100)
	PC: Pearson chi2(1) = 68.4502 Pr < 0.001 H: Pearson chi2(2) = 75.2819 Pr < 0.001					

PC- primary care, but only German speaking participants. H-hospital participants. n = number of participants. PC Total, n = 834; H-Total, n = 1,140. Primary care data [18].

^a^ For the variable training status, there were 4 possible answers (yes, no, I am in training now, I am thinking about it), but only the responses for “yes” and “no” are displayed. The group “I am in training now” was added to the “yes”. The group “I am thinking about it” to “no”.

Panels A+B show the association between training status and influenza / COVID-19 vaccination uptake, detailed by healthcare settings. Panels C+D show the association between prior influenza / COVID-19 vaccination (2020/21) and intention to get vaccinated against the influenza in the 2021/22 season, detailed by healthcare settings. Panel E shows the association between influenza and COVID-19 vaccination uptake, detailed by healthcare setting.

This difference was also seen among those vaccinated against COVID-19 during 2020/21 (Table 5). 62.0% of HCWs in the PC that were vaccinated intended to be vaccinated against influenza during 2021/22 compared to 9.8% of unvaccinated (chi2(1) = 73.7844, Pr < 0.001); 37.7% of those in the hospital setting intended to be vaccinated against influenza, compared to 0.8% of those not vaccinated (chi2(2) = 100.2009, Pr < 0.001).

### The Impact of Vaccination Training on Vaccination Status and Recommendation

Of the HCWs participating in the surveys, overall 65.5% of HCWs in the PC and 49.0% in the hospital settings stated that they received training in vaccination (Tables 1–3); compared to those in the hospital, those who were 31 years and older working in the PC cited more often that they have received vaccination training (Table 3). Table 5 also shows that vaccination training significantly increased vaccination rates for both infectious diseases among HCWs, with a clear difference in the PC setting: 70.2% of those who received vaccination training were also vaccinated against the influenza compared to 39.5% of those without training (Pearson chi2(1) = 72.8826 Pr < 0.001); 52.6% of HCWs in hospital setting with training were also vaccinated, compared to 47.4% without training (chi2(1) = 3.5036, Pr =0.061). 94.0% HCWs in PC with training were vaccination against COVID-19 compared to 90.0% without training (chi2(1) = 4.3908, Pr =0.036). In the hospital setting, 87.7% with training were vaccinated, compared to 90.5% without training (chi2(1) = 2.4641, Pr =0.116).

HCWs with training in vaccination also recommended influenza vaccination more often: 75.8% of HCWs in PC setting with training recommended influenza vaccination, compared to 40.6% of their counterparts in the hospital setting (Table 4). Similarly, it was 85.5% and 67.8% for those with vaccination training and recommending the COVID-19 vaccination.

### Reasons for Getting Vaccinated Against Influenza and COVID-19

**FIGURE 1:**
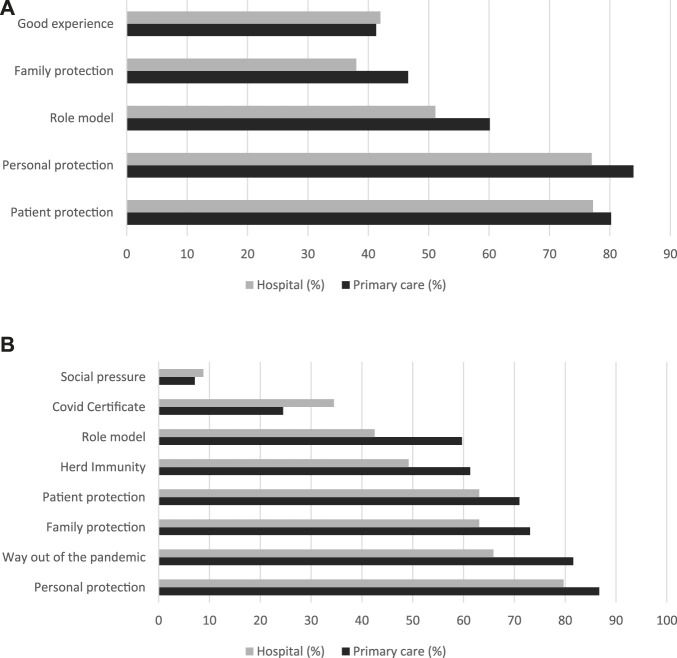
Reasons for getting vaccinated against the Influenza or COVID-19 as cited by healthcare workers, detailed by healthcare setting (Switzerland, 2021/22). Reasons cited are part of multiple response questions. Panel **(A)** Reasons for getting the influenza vaccination. Panel **(B)** Reasons for getting the COVID-19 vaccination. Primary care data originates from [18].

The distribution of the reasons for getting the influenza vaccination was similar in both settings (Figure 1). These reasons included protection of oneself, the patient and the family, being a role model and having a good experience with previous vaccinations (Figure 1A).

While personal and patient protection remain high as a reason for getting a COVID-19 vaccination, family protection increased to 73%, when referring to getting vaccinated against influenza (Figure 1B). However, the distribution of the reasons for getting a COVID-19 vaccination was different between the two healthcare settings (Figure 1B). Only 65.9% of HCWs in the hospital cited a way out of the pandemic as a reason for getting a COVID-19 vaccination, compared to 81.6% of those in PC. Similarly, herd immunity as a reason for getting vaccinated was also cited less in the hospital (49.2% vs. 61.3% PC). In contrast, 34.5% of HCWs in the hospital and only 24.5% in PC stated having a COVID-19 certificate as a reason for getting vaccinated.

## Discussion

The results of this study showed that 37% of HCWs were vaccinated against influenza and 89% against COVID-19 in the hospital, which are lower than those of the previous study where 60% and 92%–93%, respectively, were vaccinated in the PC setting nationally [18] and among German-speaking speaking HCWs. Compared with other Swiss studies, influenza vaccination rates among HCWs in both settings in the present study was higher, whereas COVID-19 rates were comparable (Table 3). Overall, HCWs demonstrated higher vaccination uptake than the general Swiss population for both influenza and COVID-19 (Table 3).

### Vaccination Status by Demographics

Although the overall vaccination rates differed between the two healthcare environments, COVID-19 vaccination rates were notably high in both PC (93%) and hospital (89%) settings. This positive outcome indicates stronger uptake of COVID-19 vaccines among HCWs, potentially reflecting acceptance of the vaccine prophylaxis, as well as the notable push to get vaccinated in order to obtain a COVID-19 vaccine certificate during the pandemic. A significant trend was observed with COVID-19 and influenza vaccination rates increasing with age in both settings, suggesting a heightened awareness of risk during the pandemic [2, 8, 9, 18, 19].

Higher influenza and COVID-19 vaccination rates have been observed among PC physicians compared to hospital-based physicians. This can be attributed to factors such as direct patient responsibility, continuous patient interaction, and possibly more robust vaccine advocacy within PC settings [20–22]. Self-employed physicians working in the private sector face financial losses if they close their practice due to illness. Consequently, personal protection—a key motivation for receiving influenza and COVID-19 vaccinations—tends to be more pronounced in the PC settings than in hospitals [23]. Research shows that a high level of commitment and a consistent presence in daily practice not only increases income, but also promotes vaccination willingness and acceptance among physicians themselves as well as their patients [21, 24–26].

Conversely, lower influenza and COVID-19 vaccination rates, as well as lower intent to vaccinate, among nurses and MPAs in hospitals may reflect systemic issues such as vaccine accessibility, perceived risk, and possibly the influence of workplace culture on vaccine perception [21, 25–28]. Furthermore, studies have also shown that the lack of targeted educational programs addressing the specific concerns and needs of these groups may contribute to lower vaccination rates [18, 22, 28–30]. Addressing these disparities requires comprehensive strategies to improve vaccine access, enhance communication and education tailored to different groups of HCWs, and foster a supportive organizational culture [20, 29–32]. Moreover, leadership endorsement of vaccination programs, can play a pivotal role in modelling positive vaccine behavior, especially in hospital settings where hierarchical structures are pronounced [33, 34].

The distribution of different healthcare professionals, age groups and sexes affects the overall vaccination uptake in both healthcare settings. Previous studies have consistently shown that physicians have higher influenza vaccination coverage than other HCWs, while evidence on sex differences in influenza vaccination is heterogeneous and context dependent [35, 36]. In Switzerland, approximately 58% of physicians were male and 41% female [37]. As men are overrepresented in the professional group with the highest vaccination uptake, aggregated vaccination rates may therefore appear higher among men than among women [35,38]. This interpretation is consistent with a systematic review and meta-analysis showing that men generally exhibit higher intentions to receive the COVID-19 vaccine than women, an effect that was more pronounced among HCWs [38].

The average age of physicians in Switzerland varies significantly between sectors. In hospitals, the average age is 44 years, primarily because younger physicians complete their mandatory specialist training in this setting. Upon completing their training, many physicians aim to become independent and transition into private practice, where the average age is significantly higher at 55 years. Notably, a quarter of practicing physicians are aged 60 or older, reflecting the trend of extended careers in this sector [39]. By contrast, nurses and MPAs enter the workforce at a younger age due to shorter training pathways. The average and median age of nursing and caregiving staff remained fairly constant at 39 years between 2012 and 2019 [40]. MPAs, on the other hand, predominantly work in outpatient practices, reflecting the specific nature of their professional roles [41]. This demographic distribution aligns with the results of the study where the likelihood of receiving influenza and COVID-19 vaccination increased with age.

Understanding the diverse vaccination behaviors among healthcare professionals requires considering the multifaceted nature of the healthcare workforce. Studies such as those by Galanis et al. [42] and Dini et al. [43] provide insight into how the role and work environment influence vaccination attitudes among HCWs. These studies highlight differences in vaccination uptake between PC and hospital settings. Consistent with our findings, research by Schmid et al. [44] also emphasizes the influence of demographic composition on vaccination acceptance, suggesting that societal and cultural attitudes towards vaccination are significant factors.

### Influence of Vaccination Training

Our results clearly show that vaccination training significantly increases the likelihood of HCWs getting vaccinated, emphasizing the effectiveness of educational and training programs in enhancing vaccine uptake among this group. Studies by Shekhar et al. and Brinko et al. have respectively highlighted the role of healthcare providers in influencing vaccine hesitancy and the positive impact of targeted educational efforts on vaccine acceptance among HCWs [3, 45]. These findings are consistent with the observation that PC HCWs generally have higher vaccination rates, which may reflect the preventive focus of these settings [18, 46, 47].

Furthermore, the strong correlation between having previously been vaccinated against both influenza and COVID-19 and the intention to get vaccinated in the future underscores the importance of initial vaccine acceptance. This pattern is supported by national and international research, demonstrating the critical role of HCWs’ vaccination status in predicting future vaccination behaviors [18, 48].

The notable interplay between influenza and COVID-19 vaccinations suggests that the acceptance of one vaccine can positively influence acceptance of another. Several publications have explored this interrelationship, highlighting that vaccination behaviors among healthcare professionals are often shaped by an overarching trust in vaccines as a preventive measure [42, 49, 50]. Particularly, recent studies indicate that this acceptance reflects a general attitude towards vaccination that is rooted in a belief in the efficacy and value of immunization [51].

### Reasons for and Against Influenza and COVID-19 Vaccinations

Our study highlights the motivations of HCWs for recommending influenza and COVID-19 vaccinations, revealing key reasons that vary between PC and hospital settings. Between 63% and 84% of HCWs across both settings prioritized patient and personal protection [52]. PC HCW place greater emphasis on family safety for COVID-19 vaccination, possibly due to closer family connections [18, 49].

Studies have shown that physicians and other HCWs are the most trusted source of health information and vaccinations for patients [53]. The fact that 60% of physicians in the PC setting see themselves as role models for their vaccination behavior is encouraging [18]; in contrast, only 40%–50% of physicians in the hospital settings have this understanding [54]. Also notable is that 66% HCWs in hospital setting and 82% HCWs in PC view the COVID-19 vaccination as a way to end the pandemic. Likewise, 50%–60% cited accepting a COVID-19 vaccination to achieve herd immunity, with a slightly more emphasis in PC, highlighting a broader public health outlook.

Risk perception is one of the most important factors influencing vaccine acceptance [2, 3, 54]. The perception among HCWs that they are not at risk of contracting influenza and COVID-19 underscores a critical gap in their understanding of their role in disease transmission, particularly given their proximity to vulnerable populations [1, 3, 45]. Additionally, the necessity for transparent and accessible information regarding vaccine development, safety, and the innovative mRNA technology as well as a general distrust of the pharmaceutical industries, reflects broader cultural societal attitudes towards vaccination [21, 32, 46]. Gender-related concerns, such as misconceptions about fertility, further complicate acceptance, despite evidence that the COVID-19 vaccination does not impair fertility in either partner [48]. These factors suggest that vaccine hesitancy among HCWs is influenced not only by individual health beliefs, but also by wider societal dynamics and perceptions [55]. Addressing vaccine hesitancy requires educational campaigns that emphasize HCWs’ public health role, alongside interventions that consider personal beliefs and broader societal influences to enhance vaccination rates [53–55].

### Limitation

Despite using the same method of data collection and questionnaire, direct comparisons between the two settings may be limited. While data for PC was collected nationally, hospital data was limited to a single cantonal hospital in Central Switzerland. This suggests that the results may not be representative of cantonal hospitals or regions with different demographic and organizational characteristics. However, research indicates that the demographic and organizational characteristics of HCWs in Central Switzerland are representative of other regions, supporting the generalizability of the results [56]. Only HCWs in five main departments of the hospital, as described in the methods, were invited to participate, which limited access to certain professional groups or departments with potentially different attitudes and experiences. However, the selection of departments was strategic, aiming to capture the groups of HCWs most affected and most representative [51]. Moreover, as this survey was conducted exclusively in German and at a single hospital, the generalizability of the results to Swiss HCW in a hospital setting is limited. With respect to participation, the hospital response rate of 26% is comparable to that reported in similar HCW surveys and reduces concerns regarding non-response bias [57]. In contrast, a response rate could not be calculated for the PC setting because survey invitations were distributed via electronic newsletters from multiple healthcare organizations, precluding accurate estimation of the denominator as impossible to determine how many individuals received and opened the survey invitation. Despite this limitation, the predefined sample size was achieved for both studies, supporting the statistical validity of the analyses.

Cross-sectional studies are for identifying factors associated with vaccination uptake, but they do not determine causation. As participation in the survey was voluntary, it is possible that individuals with a positive attitude towards vaccinations may be more willing to participate in the survey, which could introduce selection bias. Responses are based on the participants’ memories, which can lead to inaccuracies, particularly regarding vaccination details and reasons for or against vaccination. Nevertheless, self-reported data are commonplace and accepted in epidemiological research, and similar biases can be effectively minimized [58]. Furthermore, since influenza vaccination is not routinely recorded in vaccination cards, we must rely on self-reports to determine influenza vaccination rates.

Although the questionnaire was structured and comprehensive, the predefined response options may not have fully captured the complexity of individual reasons for or against vaccination. Data collection took place at specific times, which may not have considered seasonal fluctuations and time-limited events. However, standardized questionnaire designs allow for direct comparisons, and time limitations can be minimized through broad survey windows [59].

### Conclusion

HCWs in hospital settings are less likely to be vaccinated against influenza than those in PC settings. Our results also highlight the complexity of factors influencing vaccination among HCWs, which depend on profession, vaccination history and training. Public health campaigns aiming to increase vaccination rates among HCWs emphasize the dual benefits of vaccination (protecting oneself and others), the role of HCWs as health leaders, and the wider societal impact of achieving herd immunity.

Moreover, more effective tailoring of strategies could involve addressing specific concerns and barriers that may differ between settings (e.g., PC vs. hospital) and among professional roles. For instance, using the high value placed on modelling good behavior and the collective goal of ending the pandemic could be particularly persuasive.

In conclusion, understanding the multifaceted motivations for or against vaccination among HCWs provides valuable insights for designing targeted interventions to improve vaccination rates. Such efforts are crucial for enhancing the health and safety of both HCWs and the communities they serve, particularly in the face of ongoing and future health crises. Deeper qualitative analysis through interviews or focus groups could provide more detailed insights into the attitudes and perceptions of HCWs towards vaccinations [51].
