# Supplementary material for: Influence of the Workplace on Influenza and COVID-19 Vaccination Acceptance Among Swiss Healthcare Workers During Season 2021/22
Source: Int J Public Health. 2026 May 8;71:1608922. doi: 10.3389/ijph.2026.1608922 (PMC13194077; doi:10.3389/ijph.2026.1608922)
Supplement: Supplementary file 1 [file Table1.docx]

**Supplementary Table 1**

|  | **Table 4 a**. **Table 1a**. Exploratory Analysis: Association of influenza vaccination status with various factors in  primary care (PC) versus hospital setting (H) using multivariate logistic regression (Switzerland, 2021/22) | | | | | | | | | | | |
| --- | --- | --- | --- | --- | --- | --- | --- | --- | --- | --- | --- | --- |
|  | | Responders  PC H | | Influenza Vaccination  Primary Care (PC) | | | | Influenza Vaccination  Hospital (H) | | | | |
| **Characteristics** | | n | n | Adjusted Odds Ratio | 95%CI Lower | 95%CI Upper | p-value | Adjusted Odds Ratio | 95%CI Lower | 95%CI Upper | p-value |  |
|  | |  |  |  |  |  |  |  |  |  |  |  |
| **Vaccination Training&** | |  |  |  |  |  |  |  |  |  |  |  |
| No | | 281 | 581 | Ref | - | - |  | Ref | - | - |  |  |
| Yes | | 553 | 559 | 1.7534 | 0.8679 | 3.5424 | .118 | 1.8288 | 1.3515 | 2.4746 | <.001 |  |
|  | |  |  |  |  |  |  |  |  |  |  |  |
| **Sex** | |  |  |  |  |  |  |  |  |  |  |  |
| Male | | 136 | 231 | Ref | - | - |  | Ref | - | - |  |  |
| Female | | 698 | 909 | 1.4078 | 0.6255 | 3.1686 | .409 | 0.4901 | 0.3440 | 0.6981 | <.001 |  |
|  | |  |  |  |  |  |  |  |  |  |  |  |
| **Age group** | |  |  |  |  |  |  |  |  |  |  |  |
| 16-20 | | 19 | 85 | Ref | - | - |  | Ref | - | - |  |  |
| 21-30 | | 86 | 295 | 1.7303 | 0.4966 | 6.0287 | .389 | 1.2780 | 0.6770 | 2.4126 | .449 |  |
| 31-40 | | 159 | 301 | 2.3805 | 0.7029 | 8.0620 | .163 | 1.8974 | 1.0111 | 3.5604 | .049 |  |
| 41-50 | | 229 | 218 | 3.0559 | 0.9090 | 10.2735 | .071 | 2.1436 | 1.1224 | 4.0942 | .021 |  |
| 51-60 | | 261 | 197 | 4.5236 | 1.3542 | 15.1101 | .014 | 2.5437 | 1.3266 | 4.8774 | .005 |  |
| 61+ | | 80 | 44 | 3.6189 | 0.9929 | 13.1910 | .051 | 2.6216 | 1.0706 | 6.4191 | .035 |  |
|  | |  |  |  |  |  |  |  |  |  |  |  |
| **Profession** | |  |  |  |  |  |  |  |  |  |  |  |
| Nurse | | 48 | 601 | Ref | - | - |  | Ref | - | - |  |  |
| Physician | | 152 | 218 | 5.4502 | 2.5154 | 11.9091 | <.001 | 6.4565 | 4.3393 | 9.6065 | <.001 |  |
| Pharmacist | | 283 | 12 | 3.3723 | 1.7475 | 6.5080 | <.001 | - | - | - | - |  |
| MPA | | 178 | 44 | 1.2509 | 0.6257 | 2.5007 | .527 | 0.5469 | 0.2457 | 1.2174 | .139 |  |
| Pharm.Technicians | | 50 | 4 | 1.4411 | 0.6146 | 3.3735 | .401 | - | - | - | - |  |
| Other | | 123 | 261 | 0.3712 | 0.1730 | 0.7967 | .011 | 1.5052 | 1.0501 | 2.1574 | .026 |  |
|  | |  |  |  |  |  |  |  |  |  |  |  |
| **Total** | | 834 | 1141 |  |  |  |  |  |  |  |  |  |
|  | |  |  |  |  |  |  |  |  |  |  |  |

PC= primary care. H= hospital. n=number of participants. Total in PC n=1237. Total in H n = 1140. Ref=reference. CI= confidence interval. P-values for categorical variables in the multivariate analysis were calculated using Likelihood Ratio tests. Adjusted Odds Ratio: The models were adjusted for vaccination training history, sex, age group and profession. Primary care data originates from [18]: Morgel et al. Peri-Pandemic Acceptance of Influenza and COVID-19 Vaccination by Swiss Healthcare Workers in Primary Care 2020/21: A Cross-Sectional Study. Int J Public Health. 2023 Nov 15;68.

&For the variable vaccination training, there were 4 possible answers (yes, no, I am in training now, I am thinking about it), but only the responses for “yes” and “no” are displayed. The group “I am in training now” was combined with “yes”; the group “I am thinking about it” to “no”.

|  | **Table 1b.** Exploratory Analysis: Association of COVID-19 vaccination status with various factors in primary care (PC) and hospital setting (H) using multivariate logistic regression (Switzerland, 2021/22) | | | | | | | | | | | |
| --- | --- | --- | --- | --- | --- | --- | --- | --- | --- | --- | --- | --- |
|  | | Responders  PC H | | COVID-19 vaccination  Primary Care (PC) | | | | COVID-19 vaccination  Hospital (H) | | | | |
| **Characteristics** | | n | n | Adjusted Odds Ratio | 95%CI Lower | 95%CI Upper | p-value | Adjusted Odds Ratio | 95%CI Lower | 95%CI Upper | p-value |  |
|  | |  |  |  |  |  |  |  |  |  |  |  |
| **Vaccination Training&** | |  |  |  |  |  |  |  |  |  |  |  |
| No | | 281 | 581 | Ref | - | - |  | Ref | - | - |  |  |
| Yes | | 553 | 559 | 1.7534 | 0.8679 | 3.5424 | .118 | 0.9533 | 0.6265 | 1.4504 | .823 |  |
|  | |  |  |  |  |  |  |  |  |  |  |  |
| **Sex** | |  |  |  |  |  |  |  |  |  |  |  |
| Male | | 136 | 231 | Ref | - | - |  | Ref | - | - |  |  |
| Female | | 698 | 910 | 1.4078 | 0.6255 | 3.1686 | .409 | 0.4900 | 0.2435 | 0.9858 | .045 |  |
|  | |  |  |  |  |  |  |  |  |  |  |  |
| **Age group** | |  |  |  |  |  |  |  |  |  |  |  |
| 16-20 | | 19 | 85 | Ref | - | - |  | Ref | - | - |  |  |
| 21-30 | | 86 | 295 | 0.9007 | 0.2195 | 3.6959 | .885 | 1.4961 | 0.7782 | 2.8763 | .227 |  |
| 31-40 | | 159 | 301 | 1.6431 | 0.4026 | 6.7058 | .489 | 1.2844 | 0.6716 | 2.4563 | .449 |  |
| 41-50 | | 229 | 218 | 2.0283 | 0.5072 | 8.1105 | .317 | 1.9344 | 0.9462 | 3.9546 | .076 |  |
| 51-60 | | 261 | 197 | 2.8455 | 0.7018 | 11.5367 | .143 | 2.8832 | 1.3080 | 6.3553 | .009 |  |
| 61+ | | 80 | 44 | 1.6952 | 0.3578 | 8.0328 | .506 | 6.8146 | 0.8600 | 53.9981 | .069 |  |
|  | |  |  |  |  |  |  |  |  |  |  |  |
| **Profession** | |  |  |  |  |  |  |  |  |  |  |  |
| Nurse | | 48 | 601 | Ref | - | - |  | Ref | - | - |  |  |
| Physician | | 152 | 218 | 2.6258 | 0.7623 | 9.0447 | .126 | 3.1156 | 1.4154 | 6.8583 | .005 |  |
| Pharmacist | | 283 | 12 | 3.4213 | 1.1618 | 10.0747 | .026 | - | - | - | - |  |
| MPA | | 178 | 44 | 1.2894 | 0.4300 | 3.8662 | .650 | 0.7668 | 0.3256 | 1.8063 | .544 |  |
| Pharm.Technicians | | 50 | 4 | 1.6898 | 0.4661 | 6.1261 | .425 | - | - | - | - |  |
| Other | | 123 | 261 | 1.8776 | 0.6270 | 5.6225 | .260 | 1.4456 | 0.8521 | 2.4526 | .172 |  |
|  | |  |  |  |  |  |  |  |  |  |  |  |
| **Total** | | 834 | 1141 |  |  |  |  |  |  |  |  |  |
|  | |  |  |  |  |  |  |  |  |  |  |  |

PC= primary care. H= hospital. n=number of participants. Total in PC n=1237. Total in H n = 1140. Ref=reference. CI= confidence interval. P-values for categorical variables in the multivariate analysis were calculated using Likelihood Ratio tests. Adjusted Odds Ratio: The models were adjusted for vaccination training history, sex, age group and profession. Primary care data originates from [18]: Morgel et al. Peri-Pandemic Acceptance of Influenza and COVID-19 Vaccination by Swiss Healthcare Workers in Primary Care 2020/21: A Cross-Sectional Study. Int J Public Health. 2023 Nov 15;68. &For the variable vaccination training, there were 4 possible answers (yes, no, I am in training now, I am thinking about it), but only the responses for “yes” and “no” are displayed. The group “I am in training now” was added to the “yes”. The group “I am thinking about it” to “no”.
